# Supplementary material for: Distinct catecholaminergic pathways projecting to hippocampal CA1 transmit contrasting signals during navigation in familiar and novel environments
Source: bioRxiv. 2024 Jul 24:2023.11.29.569214. Originally published 2023 Nov 30. Preprint. [Version 4] doi: 10.1101/2023.11.29.569214 (PMC10705417; doi:10.1101/2023.11.29.569214)
Supplement: 1 [file NIHPP2023.11.29.569214V4-supplement-1.pdf]

bioRxiv preprint doi: https://doi.org/10.1101/2023.11.29.569214; this version posted July 24, 2024. The copyright holder for this preprint (which was not certified by peer review) is the author/funder, who has granted bioRxiv a license to display the preprint in perpetuity. It is made available under aCC-BY 4.0 International license.

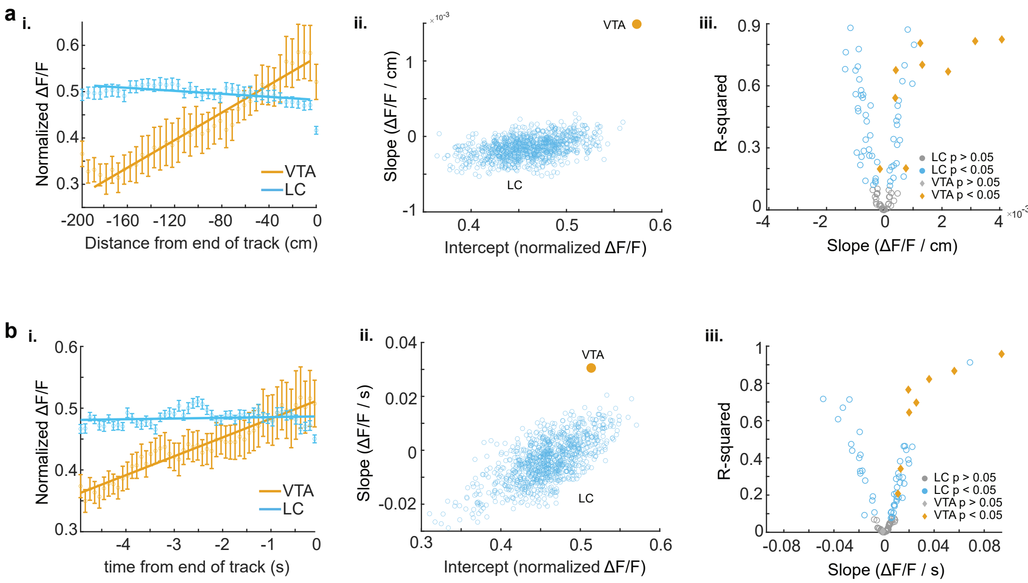

Supplementary Figure 1. a,i, Population activity $\Delta F/F\pm$ s.e.m. binned by the virtual distance to reward for VTA ROIs (orange, 200m track n = 9 ROIs in 8 mice) and LC ROIs (blue, 300m track, n = 87 ROIs from 27 sessions in 17 mice) in the familiar environment. Linear regression, F test, VTA, $P = 1.83e-28$, LC, $P = 8.77e-06$. **ii,** The LC data set was resampled 1000x using n = 9 axons to match the number of VTA Rois and the slope and intercept of the regression line were measured each time (blue dots). The VTA slope is steeper than all LC slopes indicating a stronger positive relationship between position and activity for VTA inputs.**iii,** , Linear regression of position binned activity of individual VTA (orange diamonds), and LC (blue, circles) axons. The majority (8/9) of VTA axons show a significant positive relationship with position while LC axons show both a positive (21/87 axons from 9 sessions in 8 mice) and negative (32/87 axons from 15 sessions in 9 mice) relationship.**b, i,** Same data as (a, i,) averaged by time to reward. Linear regression shows that the population of VTA axons has a significant positive relationship with time to reward. Linear regression, F test, VTA, $P = 4.44e-25$, LC,$P = 0.119$. **ii,** Resampling shows the VTA slope is above the resampled LC slopes indicating VTA ROIs have a stronger positive relationship with time to reward. **iii,** Linear regression of individual VTA and LC axons shows the majority (8/9) of VTA axons have a significant positive relationship with time to reward while LC axons show both a significant positive (31/87 axons from 14 sessions in 11 mice) and negative (17/87 axons from 7 sessions in 4 mice) relationship

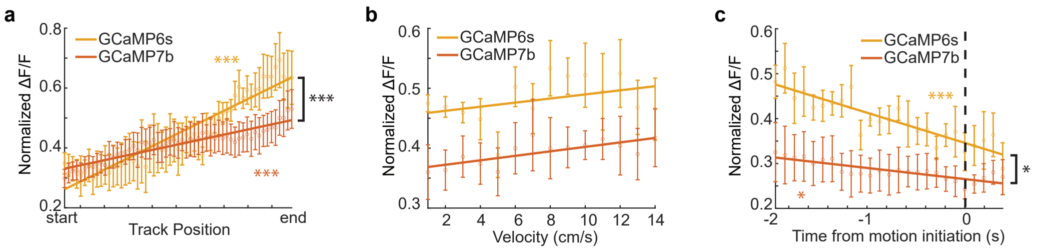

Supplementary Figure 2. a, Population average position binned $\Delta F/F\pm$ s.e.m. of VTA GCaMP6s ROIs (orange, n = 5 ROIs in 4 mice) and VTA GCaMP7b ROIs (dark orange, n = 4 ROIs, in 4 mice) in the familiar environment. Linear regression, F test, GCaMP6s, $P = 2.4264e-32$, GCaMP7b, $P = 1.9576e-14$. **b,** Same data as (a) binned by velocity. Linear regression, F test, GCaMP6s, $P = 0.21076$, GCaMP7b, $P = 0.14113$. **c,** Same data as (a) aligned to motion onset. Linear regression, F test, GCaMP6s, $P = 2.0974e-8$, GCaMP7b, $P = 0.026893$. The slopes for the two GCaMP variants were compared using a one-way ANCOVA with Tukey HSD post hoc test * $P < 0.05$, ** $P < 0.001$, *** $P < 1e-4$.

bioRxiv preprint doi: https://doi.org/10.1101/2023.11.29.569214; this version posted July 24, 2024. The copyright holder for this preprint (which was not certified by peer review) is the author/funder, who has granted bioRxiv a license to display the preprint in perpetuity. It is made available under aCC-BY 4.0 International license.

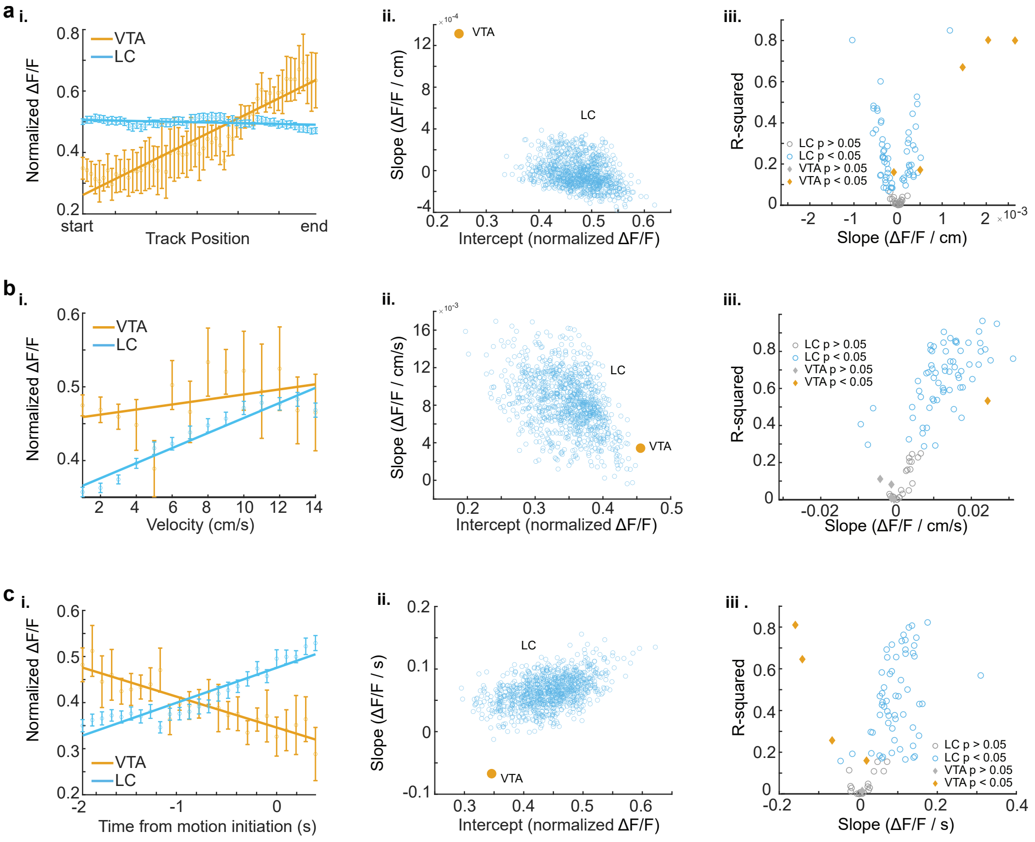

Supplementary Figure 3. a,i, Population average position binned $\Delta F/F \pm$ s.e.m. in axon-GCaMP6s expressing VTA ROIs (orange, n = 5 ROIs in 4 mice) and LC ROIs (blue, n = 87 ROIs from 27 sessions in 17 mice) . Linear regression analysis (on all data points, not means) shows that the population of VTA ROIs increase activity during approach of the end of the track while the population of LC ROIs have consistent activity throughout all positions. Linear regression, F test, VTA, $P = 2.42e-32$, LC, $P = 0.00396$. **ii,** The LC data set was resampled 1000x using n = 5 axons to match the number of VTA Rois and the slope and intercept of the regression line were measured each time (blue dots). The VTA slope is steeper than all LC slopes indicating a stronger positive relationship between position and activity for VTA inputs. **iii,** , Linear regression of position binned activity of individual VTA (orange diamonds), and LC (blue, circles) axons. The majority (4/5) of VTA axons show a significant positive relationship with position while LC axons show both a positive (25/87) and negative (37/87) relationship. **b, i,** Same data as (a, i,) binned by velocity. Linear regression shows that the population of LC ROIs have a significant relationship with velocity. Linear regression, F test, VTA, $P = 0.211$, LC,$P < 1e-68$. **ii,** Resampling shows the VTA slope is within the resampled LC slopes indicating similar relationships with velocity. **iii,** Linear regression of individual VTA and LC axons shows the majority (63/87) of LC axons have a significant positive relationship with velocity while only 1 VTA axon shows this relationship. **c, i** Same data as (a, i,) aligned to motion onset. Linear regression shows that the population of VTA axons have a negative slope prior to motion onset while LC axons have positive slope. Linear regression, F test, VTA, $P = 2.10e-081$, LC, $P = 5.51e-66$. **ii,** Resampling shows the VTA slope is negative while all resampled LC slopes are positive. **iii,** Linear regression of individual VTA and LC axons shows the majority (56/87) of LC axons have a significant positive slope prior to motion onset while the majority (3/5) of VTA axons have a negative slope.

bioRxiv preprint doi: https://doi.org/10.1101/2023.11.29.569214; this version posted July 24, 2024. The copyright holder for this preprint (which was not certified by peer review) is the author/funder, who has granted bioRxiv a license to display the preprint in perpetuity. It is made available under aCC-BY 4.0 International license.

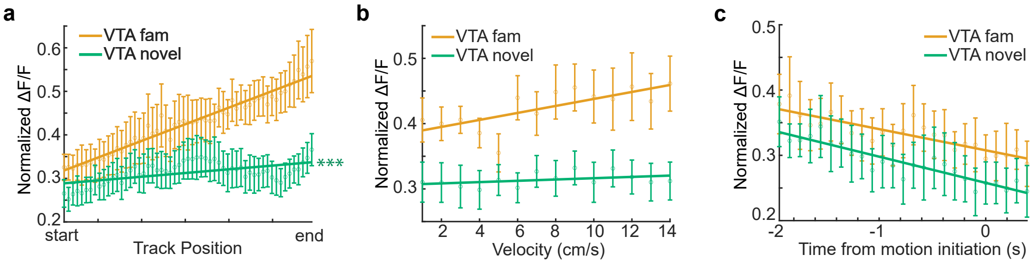

Supplementary Figure 4. a,i, Population average position binned $\Delta F/F\pm$ s.e.m. of VTA ROIs (n = 7 ROIs in 7 mice) in the familiar (orange) and novel (green) rewarded environments. Linear regression, F test, Rewarded, $P < 1e-21$, Unrewarded, $P < 0.01$. **ii,** Same data as (a,i) binned by velocity. Linear regression, F test, Rewarded, $P < 0.05$, Unrewarded, $P = 0.57$. **iii,** Same data as (a,i) aligned to motion onset. Linear regression, F test, Rewarded, $P < 0.01$, Unrewarded, $P < 0.001$. The slope of each novel measure was compared to the familiar laps using a one-way ANCOVA with Tukey HSD post hoc test. * $P < 0.05$, ** $P < 0.001$, *** $P < 1e-4$.

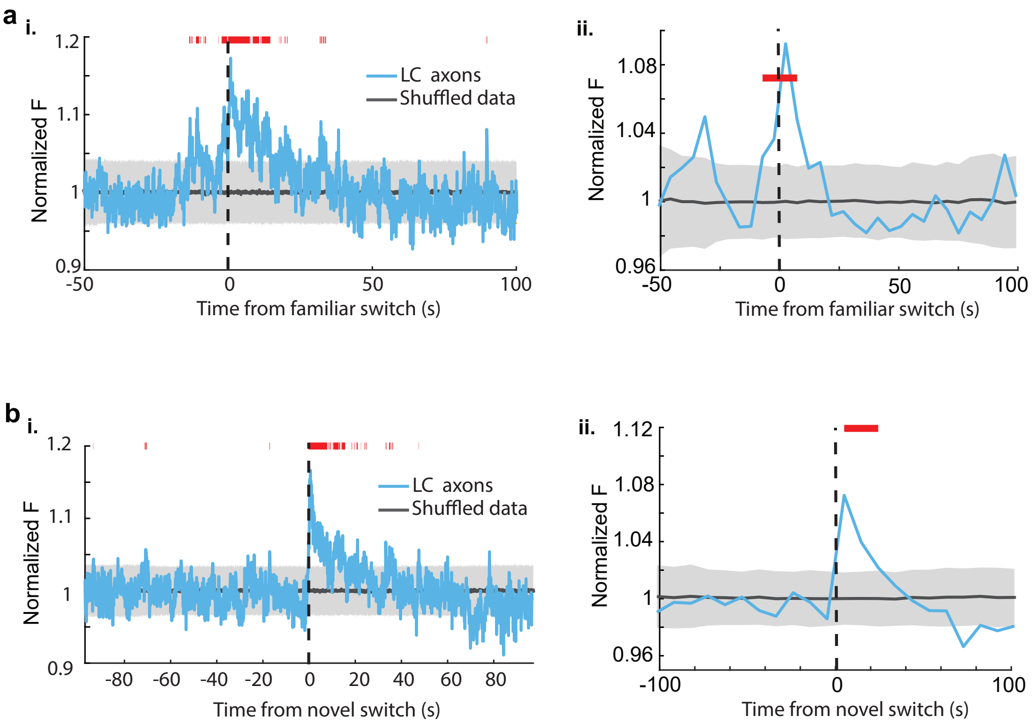

Supplementary Figure 5. a, i, Mean normalized fluorescence of LC ROIs (n = 50, 11 sessions in 9 mice) aligned to the switch from dark to the familiar environment. To define a baseline and 95% CI (gray shaded region), 1000 shuffles were created from the calcium traces and down sampled to match the sample size and averaged. This was repeated 1000 times and the mean and 95% CI of this shuffled data was determined for each frame. Red lines indicate periods where two or more consecutive frames passed above the % CI of the shuffled baseline. **a,ii,** The normalized fluorescence of all LC binned into 50 frame bins. The baseline and 95% CI (gray shaded region) was defined using the same method as in (a). Red lines indicate 2 or more consecutive bins above the baseline 95% CI. **b, i,** Mean normalized fluorescence of LC ROIs (n = 87) during immobile periods aligned to the switch from the familiar to the novel environment. The baseline and 95% CI (gray shaded region) was defined using the same method as in (a). Red lines indicate 2 or more consecutive bins above the baseline 95% CI. **b, ii** The normalized fluorescence of LC axons during immobile periods binned into 50 frame bins. The baseline and 95% CI (gray shaded region) was defined using the same method as in (a) Red lines indicate 2 or more consecutive bins above the baseline 95% CI.
